# Rapid and label-free *Listeria monocytogenes* detection based on stimuli-responsive alginate-platinum thiomer nanobrushes

**DOI:** 10.1038/s41598-022-25753-7

**Published:** 2022-12-10

**Authors:** Daniela A. Oliveira, Eric S. McLamore, Carmen L. Gomes

**Affiliations:** 1 Department of Biological and Agricultural Engineering, Texas A&M University, College Station, TX 77843 USA; 2 Agricultural Sciences, Clemson University, Clemson, SC 29631 USA; 3 Department of Mechanical Engineering, Iowa State University, Ames, IA 50011 USA

**Keywords:** Pathogens, Biosensors

## Abstract

In this work, we demonstrate the development of a rapid and label-free electrochemical biosensor to detect *Listeria monocytogenes* using a novel stimulus–response thiomer nanobrush material. Nanobrushes were developed via one-step simultaneous co-deposition of nanoplatinum (Pt) and alginate thiomers (ALG-thiomer). ALG-thiomer/Pt nanobrush platform significantly increased the average electroactive surface area of electrodes by 7 folds and maintained the actuation properties (pH-stimulated osmotic swelling) of the alginate. Dielectric behavior during brush actuation was characterized with positively, neutral, and negatively charged redox probes above and below the isoelectric point of alginate, indicating ALG-thiomer surface charge plays an important role in signal acquisition. The ALG-thiomer platform was biofunctionalized with an aptamer selective for the internalin A protein on *Listeria* for biosensing applications. Aptamer loading was optimized and various cell capture strategies were investigated (brush extended versus collapsed). Maximum cell capture occurs when the ALG-thiomer/aptamer is in the extended conformation (pH > 3.5), followed by impedance measurement in the collapsed conformation (pH < 3.5). Low concentrations of bacteria (5 CFU mL^−1^) were sensed from a complex food matrix (chicken broth) and selectivity testing against other Gram-positive bacteria (*Staphylococcus aureus*) indicate the aptamer affinity is maintained, even at these pH values. The new hybrid soft material is among the most efficient and fastest (17 min) for *L. monocytogenes* biosensing to date, and does not require sample pretreatment, constituting a promising new material platform for sensing small molecules or cells.

## Introduction

*Listeria monocytogenes* is a virulent foodborne bacterium ubiquitous in the environment (soil and water) that causes hundreds of deaths in the US annually^1,2^. Every year, nearly 48 million cases of foodborne illness are reported, nearly 19% of which are due to infection with *Listeria monocytogenes*, having potentially a $15 billion negative economic impact from healthcare costs, lost productivity, and loss of life^3,4^. With symptoms such as muscle pain, nausea, diarrhea, vomiting, and chills^1^, *L. monocytogenes* is among the deadliest of foodborne pathogens, with a fatality rate of up to 30% in high-risk individuals^5,6^. It is particularly dangerous for pregnant women, although they generally recover, their babies may not survive^2^.

*Listeria monocytogenes* is a difficult pathogen to monitor in food processing environments due to its ability to proliferate under low moisture content, high salinity conditions, or at temperatures associated with common refrigerator/freezer units^7^. A zero-tolerance rule for *L. monocytogenes* in ready-to-eat foods was implemented by the U.S. Food and Drug Administration (FDA), the United States Department of Agriculture (USDA), and the European Union (EU)^1^ compounded with the increasing number of food recalls related to *Listeria* contamination in recent years^8^ further reinforces the need for real-time pathogen detection methods that can identify contaminated food products before reaching the public.

The current state-of-the-art for detecting *Listeria* in food processing plants, including total viable counts (TVC), ELISA (enzyme-linked immunosorbent assays), and PCR (polymerase chain reaction), are susceptible to errors and false readings, and require costly laboratory settings with highly-trained personnel to complete^9,10^. Test results from these methods are severely delayed by requiring two consecutive enrichment steps (up to 48 h) to concentrate/amplify bacteria prior to a qualitative detection step^11^, because these methods lack sensitivity [~ 100 CFU mL^−1^ limits of detection (LOD)]^12,13^ and cannot directly detect low levels of pathogens in complex samples such as media, broth, or homogenized tissue.

Electrochemical sensing techniques are extensively studied for different applications due to their inherent advantages of robustness, fast response, ease of use, miniaturization, high sensitivity, low detection limits, small analyte volumes, ability to work with turbid samples, and label-free capabilities^12,14^. These properties are important to consider when analyzing environmental and food samples in a processing facility where user-friendly devices and simple sample preparation are required^15,16^. The deposition of metal nanoparticles (such as platinum) and/or polymers at the surface of electrochemical sensors is known to significantly enhance surface area and/or electrical conductivity, in addition to improving biorecognition agent immobilization, resulting in higher sensitivity, faster capture and improved detection of the target bacteria^17^. The role of platinum nanoparticles within the field of biosensing has been recently reviewed by Yu et al.^18^, who summarize their properties including high current densities, fast mass transport, and enhanced electrocatalytic properties that result in improved sensor’s key performance indicators^19–22^.

Aptamers are single-stranded oligonucleotides and, if selected properly and applied in the correct binding buffer, show high affinity toward targets^23^. Aptamers bind specifically via interactions of target(s) and exposed structures^24,25^. Aptamers have numerous advantages over antibodies, including low synthesis cost, high reproducibility, high thermal stability, and ability to modify and/or integrate various functional groups (e.g., biotinylation, thiolation, etc.)^25,26^. Comprehensive reviews on aptamer-based technologies for foodborne pathogen detection summarize recent developments in optical and electrochemical biosensors (i.e., aptasensors)^24,27,28^, including platforms that use magnetic beads conjugated to DNA aptamers and antibody-aptamer functionalized fiber-optic sensor for detection of *L. monocytogenes* cells from contaminated ready-to-eat meat products^29^. Hills et al.^30^ and Oliveira et al.^31^ reported a sensing mechanism for *L. monocytogenes* detection in food samples using the actuation of distinct chitosan-aptamer nanobrushes using the aptamer discovered by Ohk et al.^29^. Recently, Sidhu et al.^32^ developed an aptasensor using platinum interdigitated microelectrodes functionalized with this same DNA aptamer for *Listeria* spp. detection in vegetable broth and hydroponic media by incorporating the sensor in a particle/sediment trap for on-site irrigation water analysis.

The use of stimuli-responsive polymers for actuation in sensing applications has gathered increasing attention in recent years as summarized in a recent review by Hu et al.^33^. Stimuli-responsive polymers undergo major changes in solubility, volume, and/or conformation in response to external stimuli, and have been used to mimic naturally occurring responsivity to external changes on the environment that is observed in living systems^33–35^. Sodium alginate is a natural non-toxic, biodegradable, and low-cost polysaccharide containing repeating mannuronic and guluronic acids. Alginate is an anionic molecule with pKa of approximately 3.5. Above the pKa, alginate is hydrophilic and in environments that are below this pH it is hydrophobic^36,37^. Previous works have used polymer actuation for improving bacteria capture^38^ and recently Hills et al.^30^ and Giacobassi et al.^39^ studied actuation of stimuli-responsive polymers for both capture and sensing of pathogens in complex media. These studies successfully demonstrated that by controlling polymer actuation at the microscale, pathogens can be captured when the polymer is extended, and signal transduction is improved after collapsing the polymer at the sensor surface^30,39^. However, these previous studies used metals nanoparticles and polymers in a layer-by-layer deposition to produce the sensors^30,40^, requiring a multi-step fabrication process. One of the biggest challenges in these previous works was tethering the nanobrush to the surface followed by biofunctionalization, which used crosslinking techniques that were non-specific, and control of surface tethering degree versus bioreceptor immobilization was challenging.

Herein, we focus on development of a one-step co-electrodeposition of alginate thiomers and nanoplatinum as an aptasensor platform. We build on this unique alginate thiomer (ALG-thiomer) platform to develop a rapid, label-free biosensor to detect *L. monocytogenes.* Previous studies have shown co-electrodeposition of alginate and other conductive material such as graphite and MnO_2_-C^41,42^, but to the best of our knowledge, our work is the first to show simultaneous electrodeposition of ALG-thiomers and platinum for biosensing, which opens a new and exciting area of research for nano-biosensor development. We optimized the ALG-thiomer deposition conditions under different combinations of voltage, ALG-thiomer concentration and sonoelectrodeposition cycles, achieving up to sevenfold increase in the electroactive surface area for the best condition. Our results show that the highest signal-to-noise ratio was achieved when cell capture occurred in the extended brush conformation and the sensing in the collapsed brush conformation. Finally, we demonstrate that the ALG-thiomer/Pt nanobrush biosensor can selectively detect *L. monocytogenes* in a food matrix in concentrations as low as 5 CFU mL^−1^ and in the presence of other Gram-positive cells under 17 min without the need for sample preconcentration or redox labeling techniques.

## Materials and methods

### Materials and bacteria cultures

Sodium phosphate dibasic, potassium phosphate monobasic, hydroquinone (C_6_H_4_(OH)_2_, chloroplatinic acid (8% w/w), and hexaamineruthenium(III) chloride (Ru(NH_3_)_6_Cl_3_) were acquired from Sigma-Aldrich Co. (St. Louis, MO). Alumina slurry (0.05 µm) and polycrystalline diamond microsuspensions (3 µm and 1 µm) were purchased from Buehler (Lake Bluff, IL). Silver/silver chloride (Ag/AgCl) reference, platinum/iridium working (Pt/Ir, 1.6 mm dia.), and platinum auxiliary electrodes were acquired from BASinc (West Lafayette, IN). Lead acetate (30% w/v) was obtained from Fisher Scientific (Pittsburgh, PA). 1-Ethyl-3-(3-dimethylaminopropyl)carbodiimide HCl (EDC) and sulfosuccinimidyl 4-(N-maleimidomethyl)cyclohexane-1-carboxylate (Sulfo-SMCC) were acquired from Thermo Fisher Scientific (Waltham, MA). Potassium ferrocyanide trihydrate (K_4_Fe(CN)_6_·3H_2_O) was acquired from Ward’s Science (Rochester, NY). Cysteine hydrochloride monohydrate and potassium ferricyanide (K_3_Fe(CN)_6_) were acquired from J.T. Baker (Phillipsburg, NJ). Alginic acid sodium salt (low viscosity), N-Hydroxysuccinimide (NHS), platinum wire (99.95% Pt, 1.5 mm dia.), 5,5′-dithiobis-(2-nitrobenzoic acid) (Ellman’s reagent), and 2-(morpholino)ethanesulfonic acid) (MES) buffer were obtained from Alfa Aesar (Ward Hill, MA). Aptamers created by Ohk et al.^29^ that target *L. monocytogenes* membrane protein Internalin A (K_D_ = 10^3^ CFU/mL, 47 DNA bases, and 15,008 g/mol) were obtained from Gene Link Inc. (Hawthorne, NY). Yeast extract, tryptic soy broth (TSB), buffered peptone water (BPW), and tryptic soy agar (TSA), were obtained from Becton, Dickson and Company (Sparks, MD). Shelf-stable vegetable broth (UHT, ultra-high temperature processed) was acquired from a local grocery store. Dialysis tubing (DiaEasy™, 3.5 kDa MWCO) was obtained from Biovision (Milpitas, CA). Potassium chloride (KCl) and sodium chloride (NaCl) were purchased from EMD Millipore Corporation (Burlington, MA). Potassium nitrate (KNO_3_) was acquired from British Drug Houses (ON, Canada). Tris-ethylenediaminetetraacetic acid (TE) buffer (pH 7.4) was obtained from Quality Biological (Gaithersburg, MD). Tryptose phosphate broth (TPB) was obtained from HiMedia (Mumbai, India).

Bacteria cultures, *Listeria monocytogenes* (ATCC 15313) and *Staphylococcus aureus* (ATCC 25923), were acquired from American Type Culture Collection (Manassas, VA) and used as target and interferent bacteria for sensitivity and selectivity tests, respectively. Biosafety level 2 standards set by the National Institute of Health were used to conduct experiments with pathogenic microorganisms *S. aureus* and *L. monocytogenes*. All bacteria culture initially stored at − 80 °C were transferred to growth media, TPB for *L. monocytogenes* and TSB for *S. aureus*, by identical and sequential duplicate transfers and incubated under aerobic conditions for 24 h at 35 °C. *S. aureus* and *L. monocytogenes* cultures were maintained at 4 °C for up to 3 months on tryptic soy agar (TSA) and TSA with 0.6% (w/v) yeast extract (TSAYE) slants, respectively. Slant transfers to growth media were carried out to prepare bacteria cultures for analysis. Bacteria samples were enumerated by first serially diluting samples in BPW and plating on TSA and TSAYE for *S. aureus* and *L. monocytogenes*, respectively; after 48 h at 35 °C; results were recorded as CFU mL^−1^.

### Nanobrush fabrication

#### Thiomer fabrication

**Figure 1:**
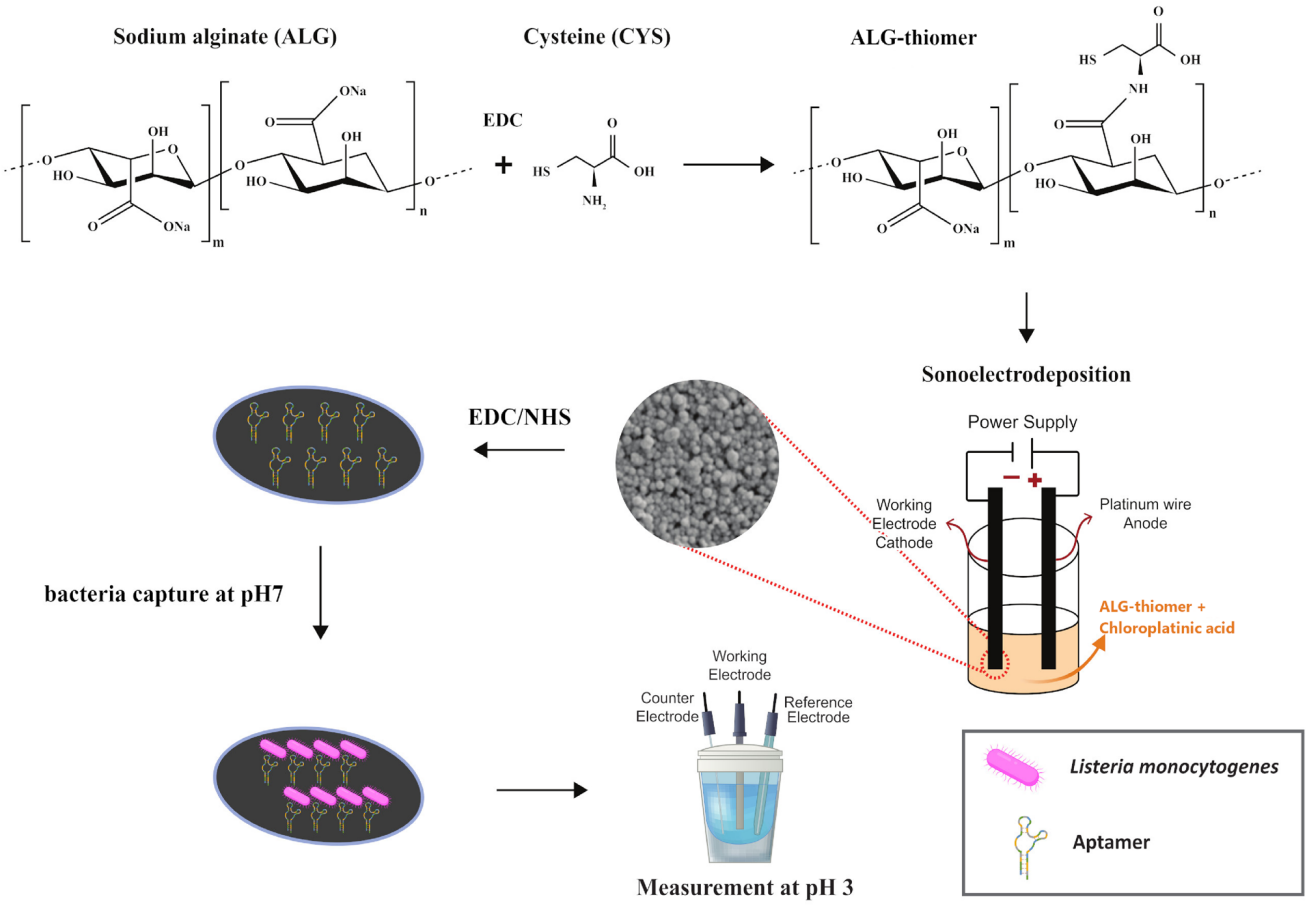
Schematic of the steps for fabrication, biofunctionalization, and sensing of the ALG-thiomer/Pt brushes functionalized with aptamer selective to *L. monocytogenes*. First, ALG-thiomers are formed based on the reaction of sodium alginate with cysteine using N-(3-dimethylaminopropyl)-N′-ethylcarbodiimide (EDC) chemistry. Then, ALG-thiomer and platinum are sonoelectrodeposited simultaneously onto the working electrode, resulting in ALG-thiomer/Pt brushes that are shown via SEM imaging. Next, the ALG-thiomer/Pt brushes were functionalized with aptamer via carbodiimide crosslinking chemistry. Sensing strategy consisted of ALG-thiomer/Pt brush actuation from collapsed to extended states based on pH changes: bacteria capture was performed at pH 7 with brushes in the extended state followed by measurement (sensing) when brushes are collapsed at pH 3.

Sodium alginate was modified to incorporate a thiol group termination by amide bond formation between carboxylic acid groups of sodium alginate and primary amino groups of cysteine using carbodiimide mediated coupling as in Jindal et al.^43^. In this method, the carboxyl groups of the alginate are activated with EDC at pH 6 for 45 min. Then, cysteine hydrochloride monohydrate was added in a weight ratio of 2:1 (alginate:cysteine) and allowed to react for 2 h at pH 4 followed by another 1 h at pH 6 (see Fig. 1 for fabrication steps). The reaction was performed under continuous stirring at room temperature.

Next, the solution was dialyzed (molecular weight cut-off 3.5 kDa) to isolate the alginate-cysteine conjugate (ALG-thiomer). The dialysis included two days against 1 mM HCl (pH 4), followed by 1 day in 1 mM HCl and 1% (w/v) NaCl, and another two days in 1 mM HCl (all the solutions were exchanged twice a day). Subsequently, the suspension was freeze-dried (− 50 °C, − 0.120 mBar, 48 h) in a Labconco FreeZone 6 unit (Labconco, Kansas City, MO, USA). ALG-thiomers were subsequently stored under nitrogen atmosphere at 5 °C until use for sensor modification.

The degree of modification achieved on the alginate was determined by quantifying the amount of thiol groups through Ellman’s analysis according to instruction from Thermo Fisher Scientific^44^. Briefly, the samples and 5,5′-dithiobis-(2-nitrobenzoic acid), namely Ellman’s reagent, were mixed in phosphate buffer containing EDTA (ethylenediamine tetraacetic acid) at pH 8 and their absorbance was measured at 412 nm against a blank control using a spectrophotometer (Genesys 10S, Thermo Scientific, Waltham, MA). A standard curve was prepared using cysteine hydrochloride monohydrate to quantify the thiol concentration (expressed as μmol of thiol groups g^−1^).

#### ALG-Thiomer/Platinum nanobrush deposition

Alginate and platinum were simultaneously deposited (Fig. 1) on the surface of platinum/iridium working electrodes (previously polished according to manufacturer instructions) using the sonoelectrodeposition method developed by Taguchi et al.^19^. In this method, a power supply connected a platinum wire (anode) and the working electrode (cathode) applied a fixed potential for 1 s followed by 1 s of sonication to the deposition solution, as alternating cycles. The deposition solution contained 0.72% (w/v) chloroplatinic acid, 0.001% (w/v) lead acetate, and ALG-thiomer at different concentrations (0.05, 0.075 and 0.1% w/v). Voltages of 1.5, 5.75 and 10 V for 60, 100 and 140 sonoelectrodeposition cycles were also evaluated to determine the optimal deposition conditions.

### Material characterization

Field emission scanning electron microscopy (SEM) was used to analyze the morphology from the best brush deposition condition based on the electrochemical experiments. After coating the electrode modified with ALG-thiomer/Pt brushes was with a 5-nm thick layer of platinum [Cressington sputter coater 208 HR (Watford, United Kingdom)], SEM images were taken by a FEI Quanta 600 FEG (Hillsboro, OR). Magnification of 10,000 and 16,000 times with accelerating voltage of 10 kV were used for the surface imaging.

The surface chemistry of the electrodes coated using the selected optimized brush deposition was analyzed by X-ray photoelectron spectroscopy (XPS), performed in an Omicron ESCA + (Scienta Omicron, Sweden) with CN10 electron gun and Mg/Al dual X-ray gun.

#### Electrochemical characterization and actuation testing

Each step of the biosensor preparation (alginate-platinum deposition, aptamer loading and bacteria detection) was electrochemically characterized using a 3-electrodes cell system (working, Ag/AgCl reference and platinum auxiliary electrodes) at room temperature using cyclic voltammetry (CV) and/or electrochemical impedance spectroscopy (EIS) methods described by Burrs et al.^17^ using a CHI 600E potentiostat/impedance analyzer. CV test was performed in a 4 mM Fe(CN)_6_^3−^ in 1 mM KNO_3_ solution with scan rates of 50, 100, 150, and 200 mV/s, 650 mV switching potential and 10 s quiet time. CV was used to obtain the electroactive surface area (ESA in cm^2^) of the electrodes via Randles–Sevcik theory using the slope of the current versus the square root of scan rate (i_p_ versus v^1/2^), as previously reported^19,45^ (see supporting information for details). The ESA values were used to determine the best deposition parameters (ALG-thiomer concentration, voltage and number of sonoelectrodeposition cycles, as described in ALG-Thiomer/Platinum nanobrsuh deposition), as well as to characterize brush actuation, and to determine aptamer loading concentration (see supporting information for details).

For the actuation tests, a negatively charged probe (KFeCN_6_^3−^), a neutral probe (C_6_H_4_(OH)_2_), and a positively charged probe Ru(NH_3_)_6_^3+^ were used. The pH of the redox probe solution was adjusted to pH 7 or pH 3 using a 1 M HCl or NaOH solution. The pH of the redox solution was monitored during the CV tests to ensure reported pH did not change by more than 0.5 pH units. Actuation tests were also carried out in the presence of a constant background *Listeria* concentration of 10^3^ CFU mL^−1^ in phosphate buffer saline (PBS) using EIS to determine the most efficient capture and measurement strategy between extended and collapsed conformation of the brushes.

#### Pathogen sensing

EIS was used to determine the limit of detection (LOD), range of detection, and sensitivity of the biosensor when exposed to bacteria at concentrations varying from 10^1^ to 10^6^ CFU mL^−1^ (standard curve). For functionalization procedure of ALG-thiomer/Pt brushes with aptamers, see supporting information for the details. The analysis was performed with initial DC potential of 0 V, AC amplitude of 100 mV and a frequency range of 1–100,000 Hz. Sensitivity testing was initially performed in PBS and then again in commercially sterile vegetable broth. From the Bode plots (impedance vs. frequency), the impedance value at fixed cutoff frequency was used to build the calibration curves, consisting of the impedance (Ohm) vs. the concentration of cells (log CFU mL^−1^). From the calibration curves (R^2^ > 0.97), the sensitivity to the target bacterium was obtained using the slope of the linear portion and then the 3σ method was used to calculate the limit of detection (LOD)^39^. Change in impedance ($\mathrm{\Delta Z}= {Z}_{bacteria}-{Z}_{no \, bacteria}$) was used to illustrate the electrodes performance independent of media. Selectivity to *L. monocytogenes* was evaluated by determining sensitivity and LOD in the presence of the identical concentration of another Gram-positive bacteria (*S. aureus*) in PBS and in sterile vegetable broth.

#### Statistical analysis

Results of independent experiments were performed at least in triplicate and expressed as mean ± standard deviation. SPSS Software was used to conduct the statistical analysis. ANOVA (one-way analysis of variance) was used to test for significance and expressed at the p < 0.05 level. Significantly different means were separated by the Tukey test.

## Results and discussion

### Nanobrush material characterization and morphology

The ALG-thiomer reaction efficiency was first evaluated by Ellman’s analysis. The result obtained was 1050 ± 200 μmol of thiol groups g^−1^ of polymer, about 3 times higher than the thiol content of 324.54 μmol g^−1^ presented by Jindal et al.^43^ with a similar procedure. In our approach, EDC (a carbodiimide compound) reacts with carboxylic acid groups of the alginate to form an active O-acylisourea intermediate that is replaced by the primary amino groups of the cysteine, forming an amide bond with the original carboxyl group (see Fig. 1). N-hydroxysuccinimide (NHS) can be included in the reaction to improve efficiency as the EDC couples NHS to carboxyls forming a NHS ester that is considerably more stable than the O-acylisourea intermediate^46^. Marcano and Sabino^47^ reported a higher value of 1939 μmol g^−1^ of thiol groups by including NHS on the activation reaction with EDC. However, this approach introduces additional functional groups which can cross link, reducing the ability to control brush actuation and introducing experimental error.

The presence of the thiol group in the Ellman’s analysis indicates that sulfur is present in the sample but does not provide information about the state of the sulfur (reactive sulfhydryl group or oxidized) as the thiol group is very susceptible to oxidation upon exposure to atmospheric oxygen with possible disulfide-like bond or cross-link (–S–S–) formation^47^.

**Figure 2:**
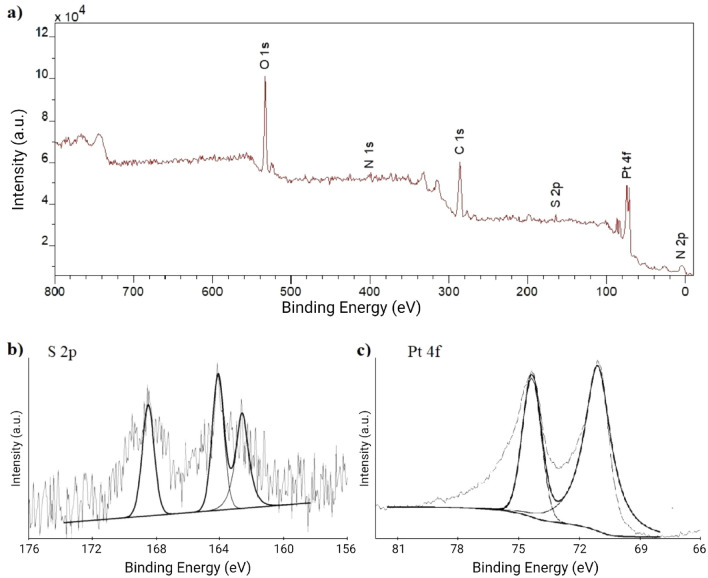
X-ray photoelectron spectroscopy (XPS) analysis. (**a**) survey spectrum, (**b**) S 2*p* spectrum, and (**c**) Pt 4*f* spectrum showing the successful co-deposition of both ALG-thiomer and Pt onto electrodes. Solid lines with smooth curves are from XPS Peak software used to identify the S 2*p* and Pt 4*f* peaks.

XPS analysis was performed to characterize the chemical bonds for the best deposition condition related to electrochemical response (see supporting information for detailed results and discussion). XPS spectrum of the ALG-thiomer/Pt brush deposition (Fig. 2a) shows the effective deposition of both ALG-thiomer and Pt components, with characteristic peaks of C 1*s*, O 1*s*, N 1*s* and S 2*p* from ALG-thiomer and Pt 4*f* from platinum. The N 1*s* peak at 399 eV fall within the range for cysteine-metal complexes^48^. The binding energy of 400 eV can be associated with the presence of amine group^40^ or with the cysteine^49^. Similarly, Wang et al.^50^ and Li et al.^51^ presented small S 2p and N 1*s* peaks on the same range as in the present work, indicating the presence of these components on carbon dots (trisodium citrate-cysteine complexes by comparing its XPS full spectrum with pure cysteine) and alkanethiol self-assembled monolayer on metallic Pt, respectively. The S 2*p* binding energy of 164 eV (Fig. 2b) is characteristic of thiol^52^ or its coordination to the metal ions^48^. The peak at 162.5 eV (S 2*p*) is also within the range expected for metal-sulfur bonds^52^. Yang and Agrios^53^ associated a doublet in the S 2p region of a Pt modified 4-mercaptobenzoic acid at slightly lower values (161.84 eV and 163.04 eV) than the obtained in the present work (162.5 eV and 164 eV) to the formation of a Pt–S bond. Castner et al.^54^ also reported the binding energy of 161.9 eV as consistent with the sulfur atoms bound to the gold surface as a thiolate species. The S 2*p* peak in the neighborhood of 168.5 eV might be due to the presence of oxidized sulfur^48,53^. The Pt *4f* spectrum (Fig. 2c) presents two peaks at 71 eV and 74.3 eV corresponding to S-bonded Pt, which is consistent with literature reports for Pt in contact with S atoms ^53,55^. Romanchenko et al.^56^ assigned the binding energy of 71.5 eV to Pt^0^ metallic nanoparticles and 74 eV to Pt(IV)-S compounds, which could also be the case in the present study as: (1) Pt could be depositing directly onto the electrode’s surface as well as binding to ALG-thiomer before depositing, and (2) the electrodeposition using chloroplatinic acid involves Pt(IV) cations and its reduction to Pt^0^^57^. Overall, the XPS spectra shown in Fig. 2 indicate bonding between the Pt nanoparticles and the S atoms in the ALG-thiomer.

**Figure 3:**
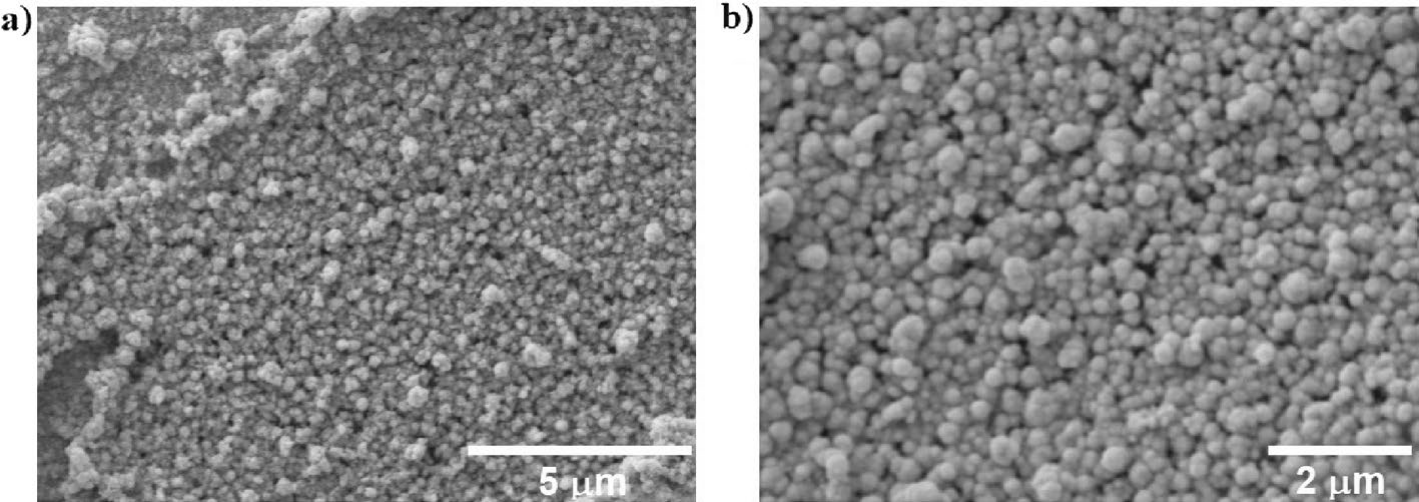
Scanning electron microscopy images of ALG-thiomer/Pt brush at 10 kV and (**a**) 10,000, and (**b**) 16,100 times magnification, respectively, showing a uniform brush formation and deposition over the electrode surface with terminal nodes ranging between 80 to 400 nm in diameter.

The morphology of the nanoplatinum and alginate brushes for the best deposition condition related to electrochemical response (see details in supporting information results and discussion) is shown in Fig. 3. The ALG-thiomer/Pt brush deposition formed juxtaposed spheroids (brush terminal nodes) with diameters ranging from 80 to 400 nm with a uniform coating with some random cauliflower shape structures distributed throughout the surface. Similar, although smoother, structure was presented by Chen et al.^42^ when simultaneously electrodepositing alginate and MnO_2_-C composite. Also, similar spheroid structure although more porous and tridimensional was shown by Giacobassi et al.^39^ with the deposition of PNIPAAm brushes over reduced graphene oxide and platinum layers. Similarly to PNIPAAm brushes described by Giacobassi et al.^39^, the electrodeposition of ALG-thiomer/Pt brushes led to homogeneous brush formation with uniform shaft and node sizes distribution. According to Taguchi et al.^19^, the pulsed sonoelectrodeposition method promotes increased mass transfer to the electrode surface, which results in uniform material deposition. The relatively large and smooth structures obtained in the present work are expected since the co-deposited ALG-thiomer complex is a significantly large polymeric material, which was also observed by Giacobassi et al.^39^. The spheroids shape increases the surface area resulting in significant increase in electrochemical surface area relative to the bare electrode (see results and discussion in supporting information). Additionally, the brush configuration in combination with brush actuation is expected to result in increased exposure of the aptamers to the medium and subsequent bacteria binding, as previously reported by Giacobassi et al.^39^ and Hills et al.^30^ (see results Sections “ALG-thiomer/Pt brush actuation and *Listeria* spp. capture” and “Biosensor performance testing”). Further investigation on the effects of the different deposition parameters on the morphology should be performed considering the importance of the morphology towards sensing performance.

### ALG-thiomer/Pt brush actuation and *Listeria* spp. capture

**Figure 4:**
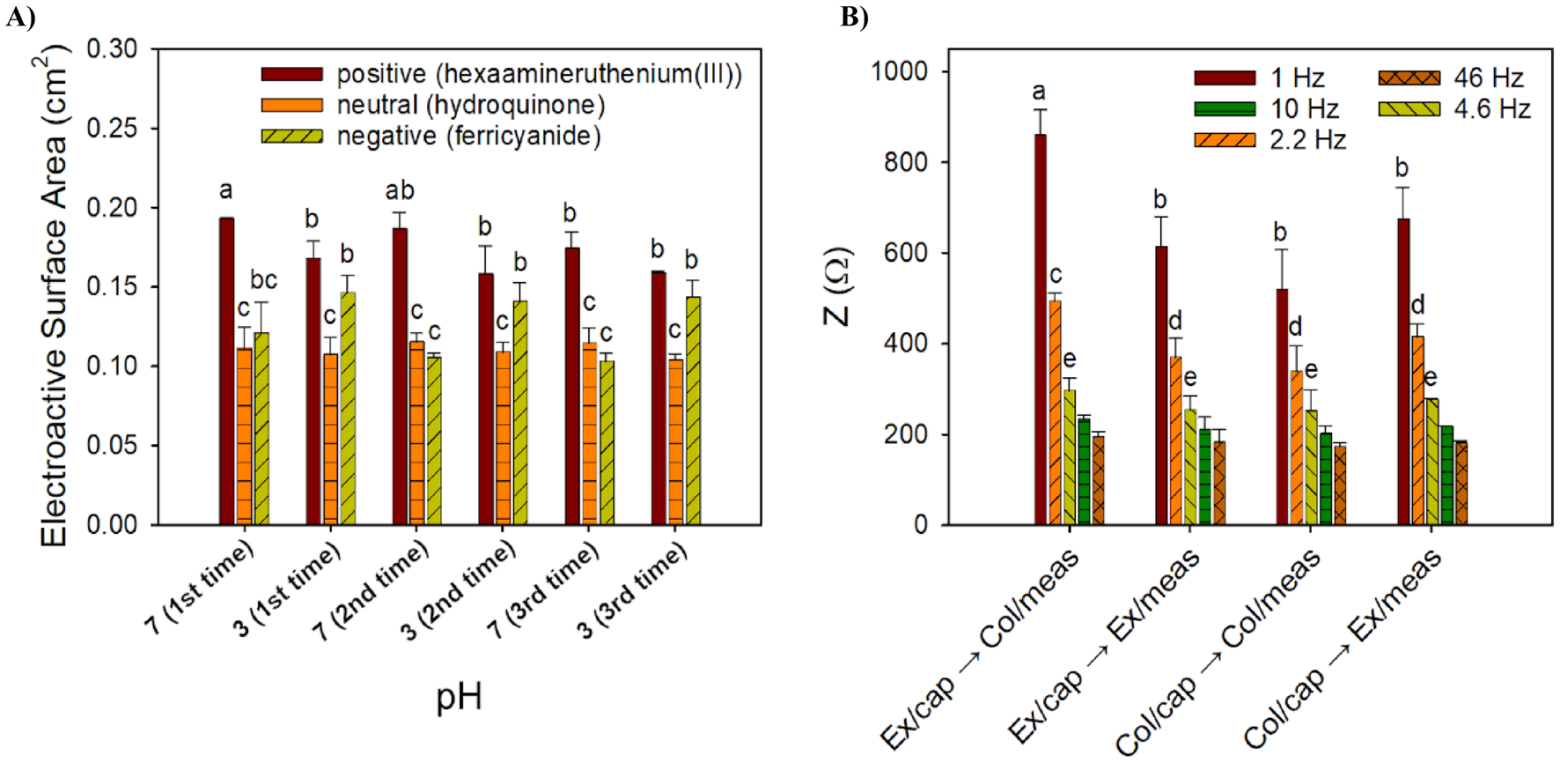
(**a**) Electrostatic interactions during three actuation cycles between pH 3 and pH 7 of the ALG-thiomer/Pt brushes with different redox probes: a positively charged probe (Ru(NH_3_)_6_^3+^, a negatively charged probe (KFeCN_6_^3−^), and a neutral probe (C_6_H_4_(OH)_2_). (**b**) Average total impedance for the ALG-thiomer/Pt brush functionalized with 400 nM aptamer and exposed to 10^3^ CFU mL^−1^ of *Listeria monocytogenes* at different cutoff frequencies for various capture/measure strategies based on actuation. “EX” refers to extended state (pH 7), “COL” refers to the collapsed brush state (pH 3), “cap” refers to cell capture and “meas” refers to measurement. Error bars denote the standard deviation of the arithmetic mean of at least three replicates; different letters represent significantly different means (p < 0.05).

Alginate is an acidic polymer that has a pKa value between 3.2 and 4 (for guluronic and mannuronic acids, respectively)^37,58^. Normally, below the pKa of the alginate carboxylic acid groups are protonized in the COOH form and the polymer is collapsed. As the pH of the solution increases, the COOH becomes ionized to COO^−^, and the resulting electrostatic repulsion among these groups causes the polymer to extend^37,59,60^. To test if the pH-stimuli actuation property of alginate was affected by its modification and to characterize the electrostatic interactions during actuation at the electrode’s surface, CV tests were carried out with three different redox probes and the ESA values were calculated (Fig. 4a). A negatively charged probe (KFeCN_6_^3−^), a neutral probe (C_6_H_4_(OH)_2_), and a positively charged probe Ru(NH_3_)_6_^3+^ were used. Moreover, CV was carried out at pH of 3 and 7, below and above the polymer’s pKa, respectively (Fig. 4a), for three repetitive cycles.

As shown in Fig. 4a, above the pKa (pH 7), electron transport decreases with the KFeCN_6_^3−^ probe due to charge repulsion/steric hindrance as the alginate carboxylate group is negatively charged; with the neutral probe (C_6_H_4_(OH)_2_) the electron transfer is less affected but still some swelling may happen due to intramolecular electrostatic repulsion among the carboxylate groups (COO^−^) of the alginate^59^; and with the Ru(NH_3_)_6_^3+^ probe, peak current and ESA increase due to electrostatic interactions. The opposite occurs below the pKa (pH 3), the electron transfer is favored with the negative probe increasing the peak current and ESA, while the peak current and ESA reduce with the positive probe. Similar behavior of ESA increasing or reducing due to electrostatic interactions or repulsion, respectively, have also been observed by Oliveira et al.^31^ and Hills et al.^30^ for chitosan actuation with the same redox probes. With repetitive actuation, peak current and ESA changed slightly (Fig. 4a) between repetitions; however, the percent change was not significant with no observable hysteresis, which indicates that ALG-thiomer/Pt brush actuation is reversible and can be repeated multiple times (at least 3 times) from collapsed to extended states. These results are an improvement from Hills et al.^30^ and Giacobassi et al.^39^ brush actuation of chitosan and PNIPAAm, respectively, that reported actuation hysteresis between repetitions.

The ALG-thiomer/Pt brush was converted into an aptasensor by functionalizing with 400 nM of amino terminated aptamer via carbodiimide cross-linking, as shown in Fig. 1 (see supporting information for details). Based on the principle that binding of target bacteria to the aptamer decreases the electron transfer at the electrode’s surface, which is measured as an increase of impedance, actuation tests were carried out with EIS to determine the optimum pH values for capturing and sensing *L. monocytogenes*. For these experiments, a constant *L. monocytogenes* concentration of 10^3^ CFU mL^−1^ in PBS was used. In Fig. 4b, the extended brush conformation at pH > 3.5 is noted as (EX), the collapsed brush conformation at pH < 3.5 is noted as (COL), the cell capture state is noted by (cap) and the electrochemical measurement step is noted by (meas). Cell capturing in the extended conformation (pH 7) and sensing at collapsed state (pH 3) provided the highest impedance values (p < 0.05) for all cutoff frequencies and, therefore, sensing efficiency (see also Bode plots in Fig. S3 in the supporting information). The optimum capture of targeted bacteria at the extended form can be related to a higher contact area with the tested solution and an increased probability of aptamer-cell interaction, compared to pH 3 at which the brushes are contracted, and the receptor binding site(s) may be inaccessible. Similar to our previous work on this capture strategy, it is likely that the cells become entangled in the polymer matrix during the sensing step, where aptamer-target binding may no longer be the mechanism for cell capture but rather entanglement during nanobrush collapse. The specificity for this detection platform is rooted in the initial affinity between aptamer and target at pH 7 (extended ALG-thiomer nanobrush), where the collapse at pH 3 likely causes secondary structure changes in the aptamer but also entraps cells in the polymer matrix.

This finding on the capture strategy is consistent with the results reported by Hills et al.^30^ and Giacobassi et al.^39^ that also presented higher sensing efficiency using the EX/cap → COL/meas strategy for pathogen detection. Additionally, the modification on the alginate with cysteine followed by the aptamer attachment, using its carbonyl groups, seems to neutralize the negative charges of alginate that would normally cause electrostatic repulsion to the highly negatively charged (under most conditions) membrane of *L. monocytogenes*^61^. This bacteria capture method is fairly simple compared to others reported in the literature such as the method by Malic et al.^62^ that developed a high gradient magnetic separation device tailored to immunomagnetic nanoparticles based on a 3D magnetic trap integrated into on a polymeric microfluidic device for magnetic capture and release of *L. monocytogenes.*

The dielectric properties of biological tissues are characterized by three dispersions including: (a) α-dispersion occurring at low frequency, associated with tissue interfaces, such as membranes; (b) β-dispersion at radiofrequency, caused by the polarization of cellular membranes, proteins and other organic macromolecules; and (c) γ-dispersion at microwave frequency, associated with the polarization of water molecules. Cell membranes act as insulating barriers at low frequencies demonstrating resistive pathways, while demonstrating high capacitance at higher frequencies. Consequently, the EIS actuation data were used to determine the cutoff frequency (CF). This analysis was conducted in a frequency range from 1 to 50 Hz, which corresponds to the alpha frequency dispersion region of biological tissues. The maximum impedance signal (p < 0.05) was observed at CF of 1 Hz (Fig. 4b) and was selected to determine key performance indicators (KPI) for the aptasensor in complex media and mixtures of bacteria in the next section.

### Biosensor performance testing

The ability of the electrodes deposited with ALG-thiomer/Pt brushes functionalized with 400 nM of amino terminated aptamer to detect *L. monocytogenes* was evaluated by EIS. See supporting information for details on the biosensor functionalization and results on optimum aptamer loading concentration onto ALG-thiomer/Pt brushes (Fig. S2). In the field of biosensors, EIS is particularly convenient to the detection of binding events on the transducer surface without the need of labels (such as fluorescence dyes, enzymes, redox or radioactive compounds)^63^. The impedimetric term arises because the interface offers (upon the occupation of the receptive centers) an electric impediment for charges to be transferred that increases as a function of target binding to the interface, which allows the label-free biosensing^64^. Impedance differences caused by the presence of bacteria (ranging from 10^0^ to 10^3^ CFU mL^−1^) were obtained using the EX/cap → COL/meas strategy (pH 7 → 3). The total test time was 17 min, which included 15 min for bacteria capture and 2 min for the EIS measurement. Bode plots are shown over a frequency range of 1 Hz to 100 kHz; insets are a zoomed in view of the lower frequency range (1–5 Hz). Based on the cutoff frequency (CF) analysis presented in Fig. 4b, all calibration curves were developed using data from Bode plots for a CF of 1 Hz.

**Figure 5:**
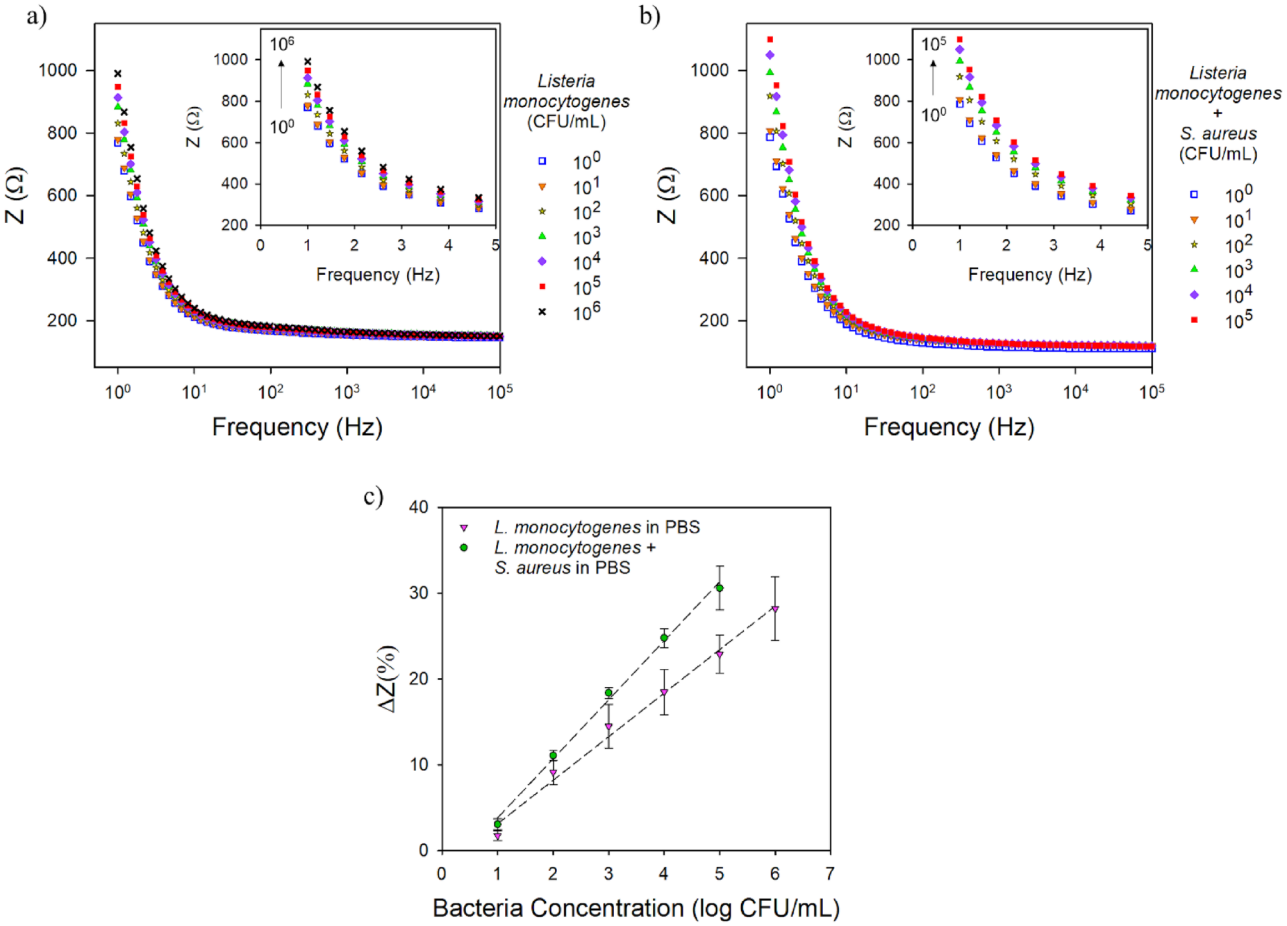
Representative Bode plots over the frequency range of 1–100,000 Hz (insets are a zoomed in view of the lower frequency range from 1 to 5 Hz) for the ALG-thiomer/Pt brush sensor functionalized with 400 nM aptamer exposed to (**a**) *L. monocytogenes* in PBS and (**b**) *L. monocytogenes* + *S. aureus* in PBS. (**c**) Calibration curves (total impedance change at 1 Hz vs. log bacteria concentration). Error bars represent the standard deviation of the arithmetic mean of at least three replicates.

Figure 5 shows ALG-thiomer/Pt/aptamer nanohybrid electrode calibrated for *L. monocytogenes* (y = 39.21x + 631.64, R^2^ = 0.99) and in the presence of *S. aureus* (y = 69.34x + 719.16, R^2^ = 0.98). The selectivity test was performed with *Staphylococcus aureus* due to its similarity to *Listeria* as Gram-positive bacteria and to both being known foodborne pathogens in addition to have similar sizes (0.5–2 µm). The addition of an equal concentration of *S. aureus* in the testing solution did not show significant interference (p > 0.05) on the LOD (4.5 ± 0.8 CFU mL^−1^ with *L. monocytogenes* alone and 5.7 ± 2.1 CFU mL^−1^ with both bacteria) indicating no cross-reaction between the sensor and *S. aureus*. The sensitivity increased (p < 0.05) from 39.21 ± 2.61 Ω/log(CFU mL^−1^) with *L. monocytogenes* alone to 69.34 ± 2.79 Ω/log (CFU mL^−1^) when *S. aureus* was also present. The linear range also changed from 10^1^ to 10^6^ CFU mL^−1^ with only *L. monocytogenes* in PBS to 10^1^–10^5^ CFU mL^−1^ in the bacteria mixture. Ohk et al.^29^ presented a LOD of 10^3^ CFU mL^−1^ using the same aptamer in a fiber-optic biosensor to detect *Listeria* spp. Recently, Oliveira et al.^31^ using the same aptamer presented similar LODs compared to the current study for *L. monocytogenes* alone in PBS and in the presence of *S. aureus*, 2.5 and 2.6 CFU mL^−1^, respectively; using actuation of chitosan/platinum brushes. While antibody sensors are reported to be prone to give false-positive reactions with *S. aureus*, which is also a protein A carrier, the aptamer used in the present work was proven to be selective to *L. monocytogenes*^29^. This aptamer targets the surface protein Internalin A which is one of the major invasion proteins involved in pathogenesis^29^. Despite the fact that this protein is structurally analogous to certain cell-wall proteins with internal repeats identified in members of the genera *Staphylococcus* and *Streptococcus*^65^, the present results corroborate that this aptamer can be selective to *L. monocytogenes*.

**Figure 6:**
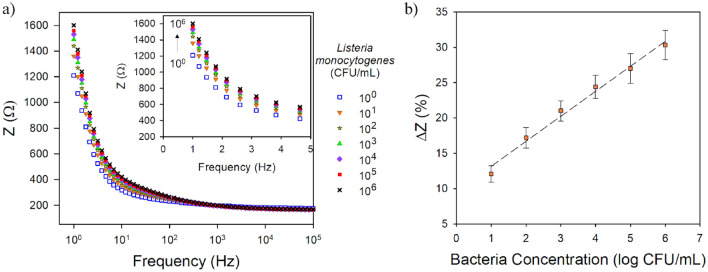
(**a**) Representative Bode plot over the frequency range of 1–100,000 Hz (insets are a zoomed in view of the lower frequency range from 1 to 5 Hz) and (**b**) calibration curve (total impedance change at 1 Hz vs. log bacteria concentration) for the ALG-thiomer/Pt brush sensor functionalized with 400 nM aptamer exposed to *L. monocytogenes* in chicken broth. Error bars represent the standard deviation of the arithmetic mean of at least three replicates.

After confirming the ALG-thiomer/Pt brush sensor ability to detect bacteria in PBS, the sensors were tested in a food product to determine its selectivity to the target bacteria as well as if the sensitivity may be affected by the interference of food components. Chicken broth (Fig. 6, y = 42.59x + 1320.93, R^2^ = 0.98) was used as it contains carbohydrates and proteins, among other components, that could interact with the biosensor through non-specific adsorption resulting in a false-positive signal. The sensitivity toward *L. monocytogenes* in chicken broth was 42.59 ± 2.35 Ω/log(CFU mL^−1^) with LOD of 4.4 ± 0.8 CFU mL^−1^, both similar (p > 0.05) to the results in PBS. These results also indicate that the aptamer is able to selectively bind to *L. monocytogenes* even in complex food matrices. Using the same aptamer, Hills et al.^30^ reported a slightly higher LOD of 9.1 CFU mL^−1^ in vegetable broth with a layer by layer reduced graphene oxide/nanoplatinum/chitosan brush electrode that actuated based on chitosan’s pH-response.

The binding effectiveness of the aptamer and its role on the biosensor performance was further evaluated by exposing the ALG-thiomer/Pt brush sensor absent of aptamers to increasing concentrations of *L. monocytogenes* in PBS (see supporting information, Fig. S4). The LOD obtained was 28.88 ± 1.31 CFU mL^−1^ with sensitivity of 23.27 ± 7.87 Ω/log(CFU mL^−1^), respectively. The performances of the sensor with aptamer previously presented were significantly (p < 0.05) better when compared to the one without aptamer. Despite of some randomly trapping of bacteria onto the nanobrushes, these LOD and sensitivity results demonstrate the aptamer’s role of selectively binding to *L. monocytogenes*, and significantly enhancing the performance of the sensor. In addition, these results further demonstrate the actuation protocol effectiveness to capture bacteria applying the ALG-thiomer/Pt brushes, which could be used as an initial step for food safety monitoring (i.e., total bacteria count).

Alginate is becoming more popular for biosensors applications due to its biocompatibility and functional groups useful for encapsulation and immobilization of biorecognition agents such for the detection of antibiotics, tumor cells, blood analysis, among others^66,67^. Some used encapsulated bacteria to monitor water toxicity^68,69^, but still, fewer reports involve bacteria detection. For instance, Kikuchi et al.^70^, used alginate to encapsulate a colorant that is cleaved by b-galactosidase enzyme from *E. coli* for its detection in breast milk, reporting a LOD of 10^2^ CFU mL^−1^ after 2 to 8 h incubation.

A compilation of current biosensors for the detection of *L. monocytogenes* in different food samples or buffers is presented in Table S1 (see supporting information). Liu et al.^71^ presented good results detecting *L. monocytogenes* in a range of 68 to 68 × 10^6^ CFU mL^-1^ by using aptamer-functionalized magnetic nanoparticles (as pre-concentration probes) and aptamer-functionalized upconversion nanoparticles (fluorescent signal probes) together; however, the procedure includes a 1 h incubation plus a pre-concentration step. Sidhu et al.^72^ proposed a platinum interdigitated array microelectrodes functionalized with the same aptamer used in the present work and reported a LOD of 5.39 CFU mL^−1^ of *Listeria* spp. in PBS also with 17 min detection. In another work, Sidhu et al.^32^ applied the same platinum interdigitated array microelectrodes functionalized with aptamers for rapid on-site flow through detection of *Listeria* spp. in irrigation water using a smartphone-based potentiostat and in flow conditions and the reported LOD was 48 CFU mL^−1^. Our sensor offers some advantages over most found in the literature, including simpler and faster fabrication with no need for cleanroom fabrication; no labeling nor bacteria pre-concentration required; and short detection time, being among the most efficient platform for *L. monocytogenes* sensing to date, with low limit of detection and wide linear sensing range relevant to food safety.

## Conclusion

Detection of a specific pathogen in any real food sample is time-consuming and laborious, as there are background signals due to impurities in the sample, either from the complex multicomponent structures or the presence of other microorganisms (pathogenic and nonpathogenic) that requires pre-enrichment treatments and laboratory settings. This study reports on a sensitive, selective and simple to fabricate impedimetric aptasensors using a one-step fabrication process of alginate thiomer/platinum nanobrushes. For the first time, we show that ALG-thiomer modified with nanoplatinum and aptamers maintain stimuli-responsive properties of the alginate while also maintaining the affinity for target bacteria, *L. monocytogenes*, due to aptamer binding. Actuation of the ALG-thiomer/Pt brushes significantly improved bacteria detection due to matrix entrapment in the collapsed state. The linear sensing range between 10 and 10^6^ CFU mL^−1^ and limit of detection of 5 CFU mL^−1^ in a food sample cover the relevant levels for food safety analysis, enabling food manufacturers to reduce economic and public health implications from recalls of contaminated food. Comparing to other methods available in the literature, this sensor is among the most efficient capture mechanisms for *L. monocytogenes*, in addition to other advantages such as simple one-step fabrication, and no labeling, no pre-incubation nor concentration required and a response time of 17 min. The results presented in this study demonstrate that the developed sensor platform is suitable for its use in food safety monitoring applications. Furthermore, this ALG-thiomer platform can be further tested for detection of other foodborne pathogens or small molecules sensing, considerably advancing detection of targets in complex matrices.

## Supplementary Information

Supplementary Information.

## Data Availability

The data presented in this study are available on request from the corresponding authors.
