# Supplementary material for: Rapid and label-free Listeria monocytogenes detection based on stimuli-responsive alginate-platinum thiomer nanobrushes
Source: Sci Rep. 2022 Dec 10;12:21413. doi: 10.1038/s41598-022-25753-7 (PMC9741594; doi:10.1038/s41598-022-25753-7)
Supplement: Supplementary file 1 — Supplementary Information. [file 41598_2022_25753_MOESM1_ESM.docx]

**Supporting Information**

**Rapid and label-free *Listeria monocytogenes* detection based on stimuli-responsive alginate-platinum thiomer nanobrushes**

Daniela A. Oliveira ^1^, Eric S. McLamore ^2^, and Carmen L. Gomes ^3,*^

^1^ Department of Biological and Agricultural Engineering, Texas A&M University, College Station, TX, 77843, USA; daoliveira@tamu.edu

^2^ Agricultural Sciences, Clemson University, Clemson, SC, 29631, USA; emclamo@clemson.edu

^3^ Department of Mechanical Engineering, Iowa State University, Ames, IA, 50011, USA

^*^To whom correspondence should be addressed. E-mail: carmen@iastate.edu

**MATERIALS AND METHODS**

**Sensor Biofunctionalization**

The biofunctionalization of the ALG-thiomer/Pt brush electrodes was performed using an aptamer that targets the protein Internalin A (A8, 5’-ATC CAT GGG GCG GAG ATG AGG GGG AGG AGG GCG GGT ACC CGG TTG AT-3’, 47 mers, GeneLink, Hawthrone, NY) on *Listeria monocytogenes* membrane. For this aptamer, two different functional groups were teste at the 5’ end termination, one with thiol and one with amine. The thiol modified aptamers were first reduced using dithiothreitol (DTT) and reconstituted in Tris EDTA (TE) buffer at pH 7.4 according to protocol provided by the manufacturer (GeneLink, 2011). The thiol terminated aptamers were diluted in TE buffer and the electrodes submerged in this suspension for two hours, shaking gently. For the amine-terminated aptamers, EDC/NHS crosslinking reaction was used. The electrodes were individually placed in 500 µL of the activation solution (0.1 M MES buffer (pH 6.0,), 0.5 M NaCl, 75 µM EDC, 25 µM NHS) for 30 min at room temperature. Next, the electrodes were transferred to a TE solution containing amine-terminated aptamers for 2 hours under gentle shaking ^1,2^. The attachment of different concentrations of each aptamer were evaluated: 200, 400, 800 and 1200 nM for the amino terminated, and 400, 800, 1200 and 1600 nM for the thiol terminated aptamer.

**Electrochemical characterization**

The electroactive surface area (ESA) of the electrode was determined using the Randles-Sevcik equation (1) ^3^:

$i_{p}=(2.69x10^{5})n^{3/2}D^{1/2}CAv^{1/2}$ (Eq. 1)

where *i_p_* (ampere) is the oxidation peak obtained from the cyclic voltammogram, *n* is the number of transferred electrons in the redox reaction (equals to 1 for the Fe(CN)_6_^3-^), *D* is the diffusion coefficient (6.70 x 10^-6^ cm^2^/s), *C* is the molar concentration of the working solution (M), *A* is the electroactive surface area (ESA) of the electrode (cm^2^) and *v* is the potential scan rate (V/s). Since *n, D,* and *C* are known properties of the working solution, *A* or ESA was calculated from the slope of the Cottrell plot (*i_p_* versus *v*^1/2^) ^3^.

**RESULTS AND DISCUSSION**

**Electrodeposition optimization**

During co-deposition of ALG-thiomer and metal, Pt(IV) ions in suspension ^4^ bind the ALG-thiomer complex and still have charges to be neutralized at the cathode. The electrodeposition of alginate is normally anodic, based on dissociation of sodium alginate forming anionic alginate species followed by its neutralization into alginic acid (H-Alg) due to a localized low pH at the anode ^5–7^. On the other hand, the platinum deposition is cathodic, as the applied direct current induces migration of positively charged platinum ions and subsequent nanoparticle formation on the electrode surface ^8^. In the present work, both methods were tested, and the cathodic deposition was selected, as no Pt deposited with anodic deposition. Thereby, the incorporation of cysteine to the alginate to provide a thiol or disulfide termination facilitates simultaneous cathodic deposition, which is the root of our one-step process. As organosulfur compounds (including alkyl thiols, dialkyl disulfides, dialkyl sulfides, among others) spontaneously form monolayers on noble metal substrates ^9^, the ALG-thiomer deposition sequence would consist of ALG-thiomer binding first to Pt via thiol-metal binding followed by cathodic deposition of the ALG-thiomer/Pt complex on the electrode’s surface. The chemisorption of organosulfur compounds in gold, which is the most studied and thoroughly characterized thiolate interaction among noble metals, is estimated at ca. 40-50 kcal/mol for the Au-S homolytic bond strength ^9^. Additionally, significant studies have also been done on thiolate chemisorption on substrates of Ag, Pd, and Pt, drawing parallels with thiolate-gold systems and demonstrating their strong and spontaneous binding ^9–11^. For instance, Petrovic et al.^12^ demonstrated that the sulfur appearing as the sulfhydryl residue of the essential amino acid L-cysteine is an excellent nucleophile for platinum sulfur-containing biomolecules, being very reactive species with high affinity for the Pt. These previous studies and reviews corroborate to the cathodic deposition of ALG-thiomer/Pt observed in the present study.

For optimization of ALG-thiomer/Pt brush deposition on electrodes, three parameters were evaluated using cyclic voltammetry (CV): deposition voltage, deposition time, and polymer concentration. Figure S4a shows representative CV curves of the best condition for the ALG-thiomer/Pt brush deposition compared to the curves of the bare Pt/Ir electrode and a dropcoated ALG-thiomer electrode. All treated electrodes demonstrated quasi-reversible redox couples with well-defined redox peaks indicating that the reaction was diffusion controlled at the interface of the electrode-solution ^3^. No significant change in current peak value (p < 0.05), and consequently on ESA value was observed when ALG-thiomer was dropcoated onto the electrode’s surface, which indicates that the presence of thiol groups did not promote sufficient direct attachment of ALG-thiomer to the electrode’s surface. Figure S4b presents a comparison among ESA values with the different deposition parameters. All conditions resulted in significantly higher ESA values (p < 0.05) than the bare electrodes (0.028 ± 0.003 cm^2^), indicating that coating the electrodes with ALG-thiomer/Pt brush improves electron transport at the electrode surface. The platinum is the responsible for the improved electron transport given its high electrical conductive nature, while the alginate with its long polymeric chain and functional groups is expected to assist with the aptamer binding to the brushes and pH-responsive actuation properties (see results sections 3.3. and 3.4). The best condition (5.75 V / 140 cycles / 0.05% w/v ALG-thiomer), increased the ESA value by 7 times from the bare electrode, to 0.20 ± 0.03 cm^2^.

An increase in material deposition with increasing time was reported for both platinum ^13^ and alginate ^6^ but in both cases only one voltage was applied. Other authors also presented increasing deposition of alginate with time and current density ^7,14–16^. In the present work, the combination of voltage and deposition time presented the best effect on the brush deposition. Even though two of the conditions tested were similar (p > 0.05), 5.75 V/ 140 cycles / 0.05% (w/v) ALG-thiomer was chosen for further experiments since: 1) the response surface analysis indicated the best voltage range between 5 V and 8 V; and 2) with 10 V/ 140 cycles/ 0.075% (w/v) ALG-thiomer some “overgrowth” (buildup the deposited material outside the active working area of the electrode) was observed. Overgrowth is not desirable considering that electrochemical response is dependent on surface area. Taguchi et al. ^13^ also reported overgrowth with pure Pt sonoelectrodeposition over 180 seconds considering it to not be stable as it might flake off when immersed in solution. The same authors reported ESA value of 0.3 cm^2^ when depositing nanoplatinum at 10 V for 180 seconds using the same pulSED technique ^13^. The lower ESA value (0.20 ± 0.03 cm^2^) obtained in the present work, despite the longer deposition time (280 seconds), is probably due to steric hindrance to more platinum deposition caused by the presence of the ALG-thiomer material in addition to alginate being a non-conductive material. Besides the steric hindrance, a direct comparison between the sole deposition of platinum or alginate to this work might not be valid as this was a simultaneous deposition of both, done cathodically, while sole alginate electrodeposition is usually anodic, and the alginate was modified to incorporate cysteine for a thiol termination.


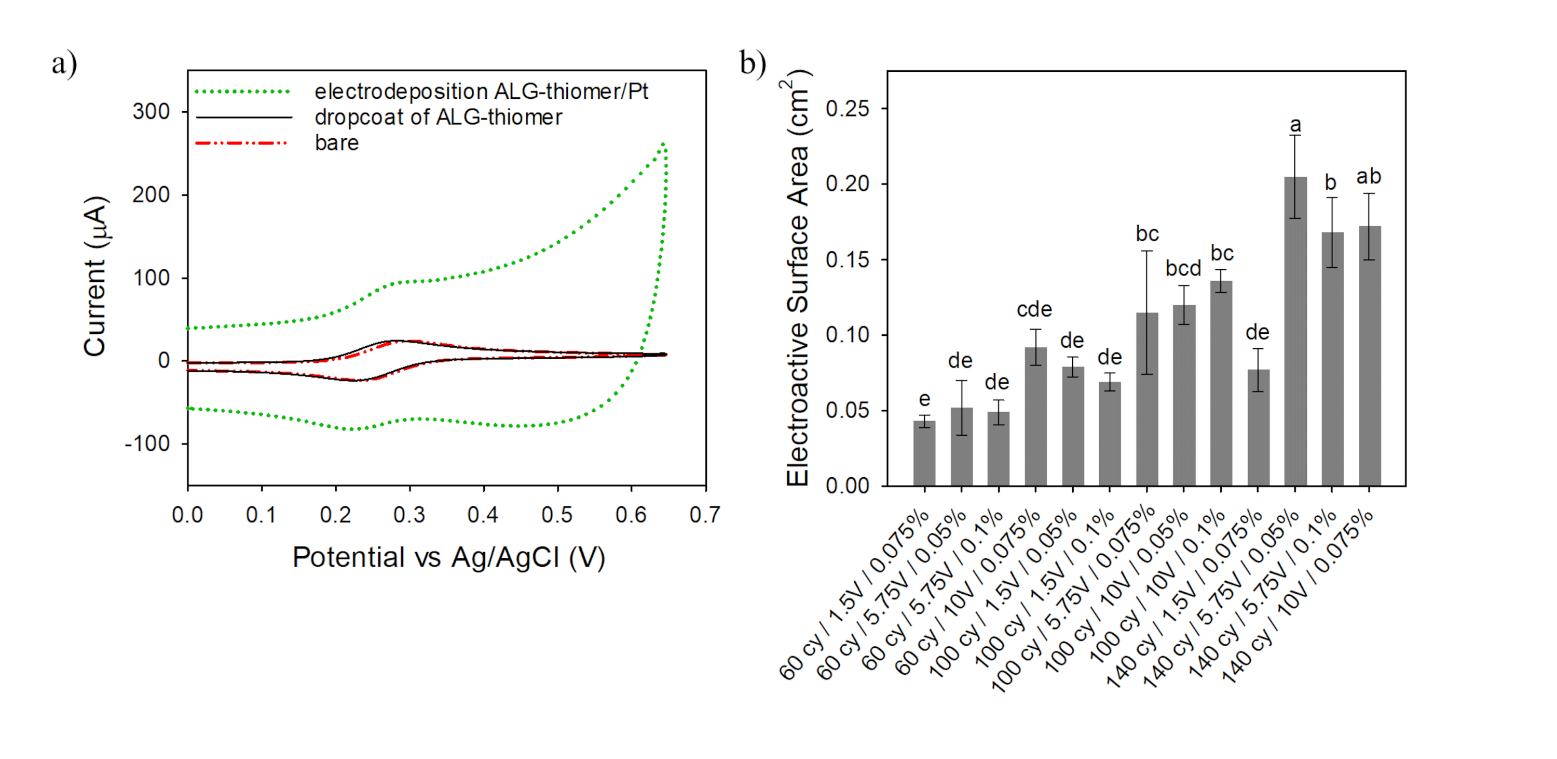


**Figure S1.** (a) Representative cyclic voltammetry curves at 100 mV/s scan rate of bare Pt/Ir, ALG-thiomer dropcoated, and ALG-thiomer/platinum brush electrodeposition (best condition: 5.75 V/ 140 cycles / 0.05% ALG-thiomer). (b) Average electroactive surface area for various ALG-thiomer/platinum brush deposition conditions with different voltages (V), number of cycles (cy)and ALG-thiomer concentration (% w/v). CV was performed using 4 mM K_4_FeCN_6_ as the redox probe (pH ~7). Error bars denote the standard deviation of the arithmetic mean of at least three replicates; different letters represent significantly different means (p < 0.05).

**Biofunctionalization optimization**

Aptamer loading onto ALG-thiomer/Pt brush electrodes was optimized based on ESA calculated from voltammograms in 4 mM K_4_FeCN_6_ at pH 7. As expected, the aptamer caused a decrease in ESA due to steric hindrance (Figure S2). It has been shown that DNA acts as an insulator ^17–19^. This effect caused by aptamer attachment in the ESA was also observed by Hills et al. ^20^. The electrodes functionalized with the thiol terminated aptamer presented similar (p > 0.05) ESA percentage change for the three lowest loading concentrations, and 800 nM (highest numerical value) was chosen for further tests with bacteria. However, the performance of the electrodes functionalized with the thiol terminated aptamer was highly inconsistent and with poor LOD (21.91 ± 1.24 CFU mL^-1^) on the preliminary tests with bacteria, consequently this aptamer was not used for further experiments. For the amino terminated aptamer, despite no significant difference (p > 0.05) between 200 nM and 400 nM loading concentration, 400 nM was chosen for better comparison with previous results from our group when working with alginate. Moreover, aptamer packing density and steric hindrance play an important role in sensing performance ^21^ and based on Figure S2, it seems that above 400 nM loading the electrodes have reached a saturation point.


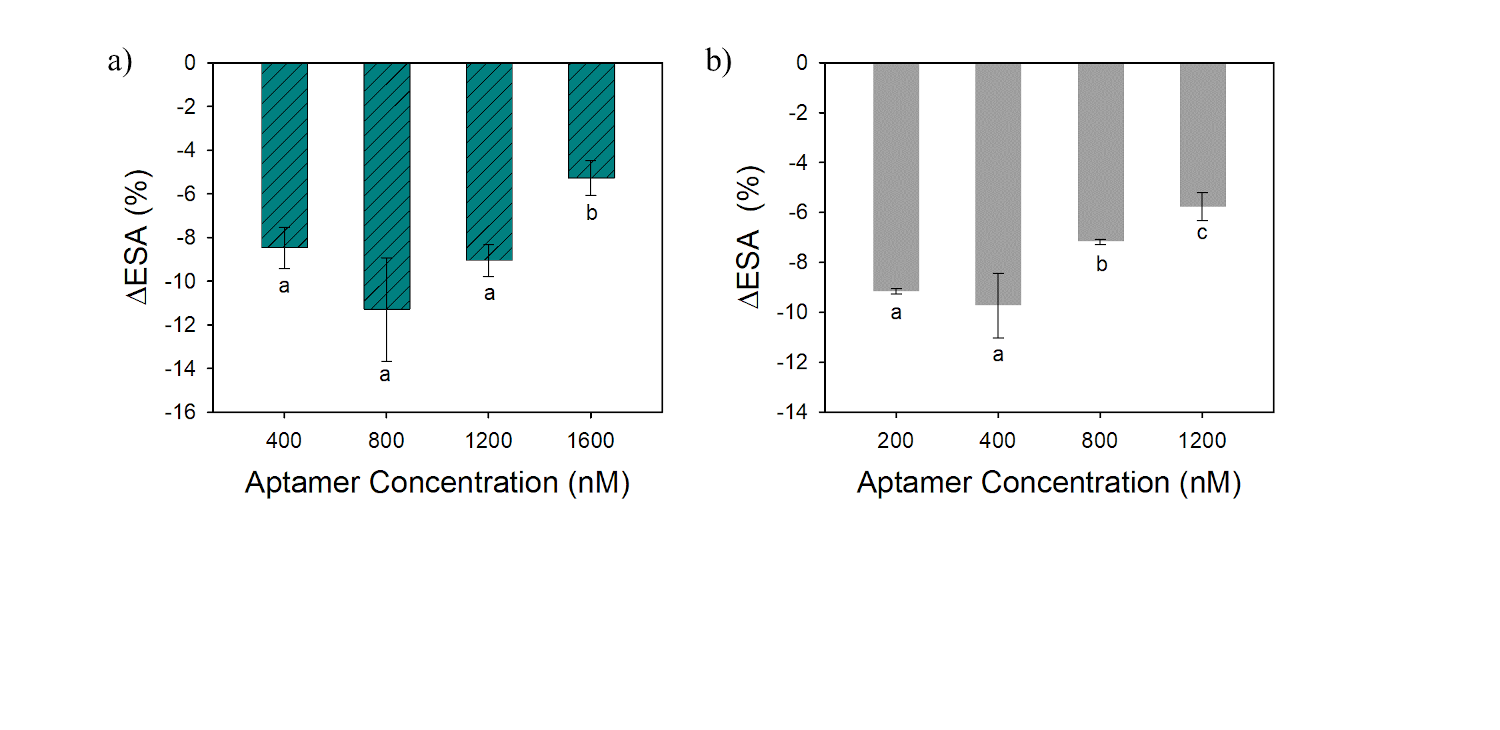


**Figure S2.** Electroactive surface area change (%) for the ALG-thiomer/platinum brush at different loading concentrations of a) thiol terminated aptamer, and b) amine terminated aptamer. Based on these results 800 nM of the thiol terminated aptamer and 400 nM of the amine terminated aptamer were used on further experiments with bacteria. Error bars denote the standard deviation of the arithmetic mean of at least three replicates; different letters represent significantly different means (p < 0.05).


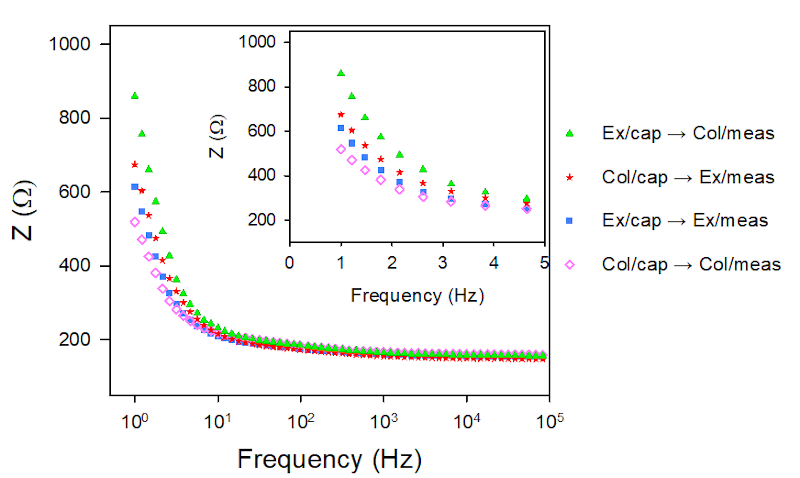


**Figure S3.** Representative bode plot for different actuation strategies for bacteria capture and sensing over the frequency range of 1-100,000 Hz (insets are a zoomed in view of the lower frequency range from 1-5 Hz) using the ALG-thiomer/platinum brush sensor conjugated with 400 nM aptamer tested with to 10^3^ CFU mL^-1^ of *L. monocytogenes*. “EX” refers to extended state (pH 7), “COL” refers to the collapsed brush state (pH 3), “cap” refers to cell capture and “meas” refers to measurement.


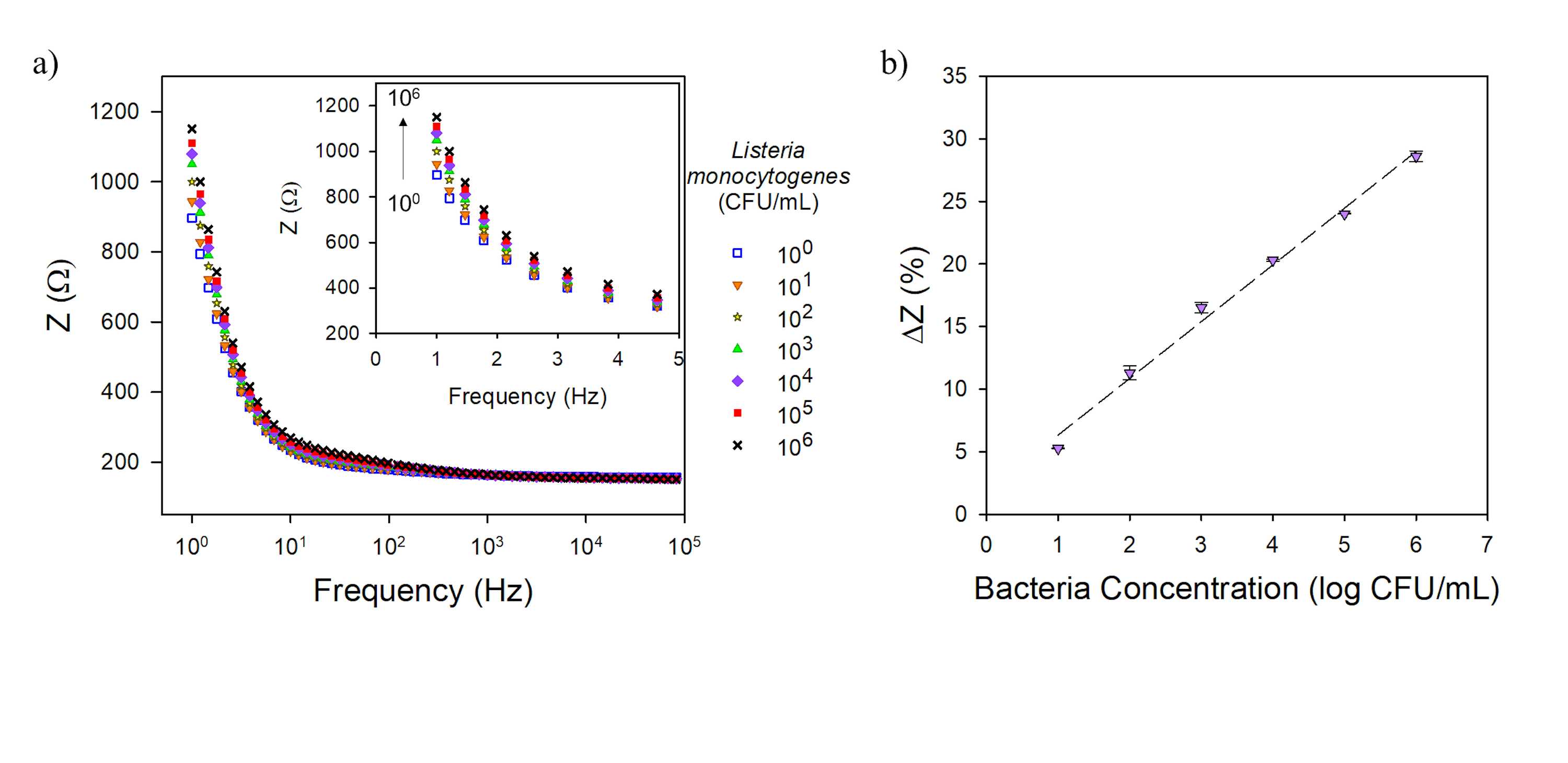


**Figure S4**. a) Representative Bode plot over the frequency range of 1-100,000 Hz (insets are a zoomed in view of the lower frequency range from 1-5 Hz) and b) Calibration curve (total impedance change at 1 Hz vs. log bacteria concentration) of the ALG-thiomer/platinum brush sensor without conjugation with aptamer tested with *L. monocytogenes* in PBS. Error bars denote the standard deviation of the arithmetic mean of at least three replicates.

**Table S1.** Comparison of recently published reports on *L. monocytogenes* detection using various transducers*.*

| **Biorecognition agent and platform** | **Detection mode** | **Test medium** | **Detection range* (CFU mL^-1^)** | **Response time (min)** | **Reference** |
| --- | --- | --- | --- | --- | --- |
| ALG-thiomer/Pt +  aptamer | Impedimetric | PBS | 4.5 - 10^6^ | 17 | This work |
| ALG-thiomer/Pt +  aptamer | Impedimetric | PBS +  *S. aureus* | 5.7 - 10^5^ | 17 | This work |
| ALG-thiomer/Pt +  aptamer | Impedimetric | Chicken  broth | 4.4 - 10^6^ | 17 | This work |
| ALG-thiomer/Pt  (no aptamer) | Impedimetric | PBS | 28.9 - 10^6^ | 17 | This work |
| rGO-nPt + CHI +  aptamer | Impedimetric | Vegetable  broth | 9.1 - 10^2^ | 17 | Hills et al. ^20^ |
| rGO-nPt + CHI +  aptamer | Impedimetric | PBS | 3 - 10^7^ | 17 | Hills et al. ^20^ |
| rGO-nPt + CHI +  antibody | Impedimetric | Vegetable  broth | 15.6 - 10^4^ | 17 | Hills et al. ^20^ |
| Pt-IDE + aptamer | Impedimetric | PBS | 5.4 - 10^6^ | 17 | Sidhu et al. ^22^ |

**Table S1.** Continued.

| **Biorecognition agent and platform** | **Detection mode** | **Test medium** | **Detection range* (CFU mL^-1^)** | **Response time (min)** | **Reference** |
| --- | --- | --- | --- | --- | --- |
| UCNP/aptamer + MNP/aptamer | Fluorescence | PBS | 68 - 68×10^6^ | > 60 | Liu et al. ^23^ |
| AIE-NP + MNP +  aptamer + antibody | Fluorescence | PBS | 10 - 10^6^ | 90 | Guo et al. ^24^ |
| GNP + antibody | Dynamic light scattering | PBS | 35 - 10^3^ | 120 | Huang et al.^25^ |
| TiO_2_ nanowire +  antibody | Impedimetric | BHI growth medium | 10^2^ – 10^7^  (non-linear) | 60 | Wang et al. ^26^ |

* Includes the lower limit of detection (LOD)

rGO-nPt + CHI: reduced graphene oxide/nano-platinum + chitosan

Pt-IDE: platinum interdigitated array microelectrodes

UCNP: upconversion nanoparticles

MNP: magnetic nanoparticles

AIE-NP: aggregation-induced emission nanoparticles

**References**

1. Balamurugan, S., Obubuafo, A., Soper, S. A. & Spivak, D. A. Surface immobilization methods for aptamer diagnostic applications. *Anal. Bioanal. Chem.* **390**, 1009–1021 (2008).

2. Jantra, J. *et al.* Real-time label-free affinity biosensors for enumeration of total bacteria based on immobilized concanavalin A. *J. Environ. Sci. Heal. - Part A Toxic/Hazardous Subst. Environ. Eng.* **46**, 1450–1460 (2011).

3. Vanegas, D. C. *et al.* A comparative study of carbon-platinum hybrid nanostructure architecture for amperometric biosensing. *Analyst* **139**, 660–7 (2014).

4. Yasin, H. M., Denuault, G. & Pletcher, D. Studies of the electrodeposition of platinum metal from a hexachloroplatinic acid bath. *J. Electroanal. Chem.* **633**, 327–332 (2009).

5. Wang, Z. *et al.* Electrodeposition of alginate/chitosan layer-by-layer composite coatings on titanium substrates. *Carbohydr. Polym.* **103**, 38–45 (2014).

6. Cheong, M. & Zhitomirsky, I. Electrodeposition of alginic acid and composite films. *Colloids Surfaces A Physicochem. Eng. Asp.* **328**, 73–78 (2008).

7. Cheng, Y. *et al.* Mechanism of anodic electrodeposition of calcium alginate. *Soft Matter* **7**, 5677–5684 (2011).

8. Grainger, S. & Blunt, J. *Engineering Coatings: Design and Application*. (Abington Publishing, 1998).

9. Sapsford, K. E. *et al.* Functionalizing nanoparticles with biological molecules: Developing chemistries that facilitate nanotechnology. *Chem. Rev.* **113**, 1904–2074 (2013).

10. Li, Z., Chang, S. C. & Williams, R. S. Self-assembly of alkanethiol molecules onto platinum and platinum oxide surfaces. *Langmuir* **19**, 6744–6749 (2003).

11. Love, J. C., Estroff, L. A., Kriebel, J. K., Nuzzo, R. G. & Whitesides, G. M. *Self-assembled monolayers of thiolates on metals as a form of nanotechnology*. *Chemical Reviews* **105**, (2005).

12. Petrovic, B. V, Djuran, M. I. & Bugarcic, Z. D. Binding of platinum(II) to some biologicaly important thiols. *Met. Based. Drugs* **6**, 355–360 (1999).

13. Taguchi, M. *et al.* PulSED: pulsed sonoelectrodeposition of fractal nanoplatinum for enhancing amperometric biosensor performance. *Analyst* **141**, 3367–78 (2016).

14. Wan, W., Dai, G., Zhang, L. & Shen, Y. Paper-based electrodeposition chip for 3D alginate hydrogel formation. *Micromachines* **6**, 1546–1559 (2015).

15. Ozawa, F., Ino, K., Takahashi, Y., Shiku, H. & Matsue, T. Electrodeposition of alginate gels for construction of vascular-like structures. **115**, 459–461 (2013).

16. Kingsley, D. M., Capuano, J. A. & Corr, D. T. On-Demand Radial Electrodeposition of Alginate Tubular Structures. *Biomater. Sci. Eng.* **5**, 3184–3189 (2019).

17. Zhang, Y., Austin, R. H., Kraeft, J., Cox, E. C. & Ong, N. P. Insulating Behavior of λ-DNA on the Micron Scale. *Phys. Rev. Lett.* **89**, 2–5 (2002).

18. Gómez-Navarro, C. *et al.* Contactless experiments on individual DNA molecules show no evidence for molecular wire behavior. *Proc. Natl. Acad. Sci.* **99**, 8484–8487 (2002).

19. Bockrath, M. *et al.* Scanned Conductance Microscopy of Carbon Nanotubes and λ -DNA. *Nano Lett.* **2**, 187–190 (2002).

20. Hills, K. D., Oliveira, D. A., Cavallaro, N. D., Gomes, C. L. & McLamore, E. S. Actuation of chitosan-aptamer nanobrush borders for pathogen sensing. *Analyst* **143**, 1650–1661 (2018).

21. White, R. J., Phares, N., Lubin, A. A., Xiao, Y. & Plaxco, K. W. Optimization of Electrochemical Aptamer-Based Sensors via Optimization of Probe Packing Density and Surface Chemistry. *Langmuir* **24**, 10513–10518 (2008).

22. Sidhu, R. *et al.* Impedance biosensor for the rapid detection of Listeria spp. based on aptamer functionalized Pt-interdigitated microelectrodes array. in *SPIE* **9863**, 98630F (2016).

23. Liu, R. *et al.* Development of a fluorescence aptasensor for rapid and sensitive detection of Listeria monocytogenes in food.pdf. *Food Control* **122**, 107808 (2021).

24. Guo, Y. *et al.* A novel fluorescence method for the rapid and effective detection of Listeria monocytogenes. *Analyst* **145**, 3857–3863 (2020).

25. Huang, X. *et al.* Gold nanoparticle-based dynamic light scattering immunoassay for ultrasensitive detection of Listeria monocytogenes in lettuces. *Biosens. Bioelectron.* **66**, 184–190 (2015).

26. Wang, R., Ruan, C., Kanayeva, D., Lassiter, K. & Li, Y. TiO 2 Nanowire Bundle Microelectrode Based Impedance Immunosensor for Rapid and Sensitive Detection of Listeria monocytogenes 2008. *Nano Lett.* **8**, 2625–2631 (2008).
